# Matricellular proteins of the Cyr61/CTGF/NOV (CCN) family and the nervous system

**DOI:** 10.3389/fncel.2015.00237

**Published:** 2015-06-24

**Authors:** Anna R. Malik, Ewa Liszewska, Jacek Jaworski

**Affiliations:** Laboratory of Molecular and Cellular Neurobiology, International Institute of Molecular and Cell BiologyWarsaw, Poland

**Keywords:** nervous system, matricellular proteins, extracellular matrix, signal transduction, CCN

## Abstract

Matricellular proteins are secreted proteins that exist at the border of cells and the extracellular matrix (ECM). However, instead of playing a role in structural integrity of the ECM, these proteins, that act as modulators of various surface receptors, have a regulatory function and instruct a multitude of cellular responses. Among matricellular proteins are members of the Cyr61/CTGF/NOV (CCN) protein family. These proteins exert their activity by binding directly to integrins and heparan sulfate proteoglycans and activating multiple intracellular signaling pathways. CCN proteins also influence the activity of growth factors and cytokines and integrate their activity with integrin signaling. At the cellular level, CCN proteins regulate gene expression and cell survival, proliferation, differentiation, senescence, adhesion, and migration. To date, CCN proteins have been extensively studied in the context of osteo- and chondrogenesis, angiogenesis, and carcinogenesis, but the expression of these proteins is also observed in a variety of tissues. The role of CCN proteins in the nervous system has not been systematically studied or described. Thus, the major aim of this review is to introduce the CCN protein family to the neuroscience community. We first discuss the structure, interactions, and cellular functions of CCN proteins and then provide a detailed review of the available data on the neuronal expression and contribution of CCN proteins to nervous system development, function, and pathology.

## Introduction

Matricellular proteins are secreted proteins that exist at the border of cells and the extracellular matrix (ECM). However, instead of playing a role in structural integrity of the ECM, these proteins, that act as modulators of various surface receptors, have a regulatory function and instruct a multitude of cellular responses. Thrombospondin-1 (TSP-1), SPARC, and Tenascin-C (TN-C) are considered canonical matricellular proteins, however numerous new ones have been described. Among them are members of the Cyr61/CTGF/NOV (CCN) protein family. Over the last two decades neurobiologist have started to appreciate role of ECM in the nervous system development, physiology and pathology. Accordingly, important contributions of canonical matricellular proteins were described. In contrast, the CCN proteins, although expressed in the nervous system, received relatively little attention in this context. Instead major research efforts focused on their importance for cardiovascular system and skeleton development as well as their links with cancer. Yet, over last few years evidence has accumulated that at least some of CCN family members play important role in the nervous system, especially during development. Thus, the major aim of this review is to introduce the CCN protein family to the neuroscience community. We first briefly introduce matricellular proteins in general. Next, we provide basic facts about structure, interactions, and cellular functions of CCN proteins that are needed as a background to understand the involvement of these proteins in the nervous system. The following parts of this article contain a detailed review of the available data on the neuronal expression and contribution of CCN proteins to nervous system development, function, and pathology.

## Matricellular Proteins

The idea of matricellular proteins arose almost 20 years ago with the identification of pericellular matrix proteins, which were shown to not be directly involved in structuring the ECM but rather regulate cell function (Bornstein, 1995; Bornstein and Sage, 2002; Murphy-Ullrich and Sage, 2014). TSP-1, SPARC, and TN-C became prototypic matricellular proteins, and their properties served as selection criteria for the subsequent identification of other matricellular proteins (Bornstein and Sage, 2002; Murphy-Ullrich and Sage, 2014). Specifically, matricellular proteins should: (i) be highly expressed during development or upon injury; (ii) modulate cell-matrix interactions; (iii) bind to cell surface receptors, the ECM, growth factors, cytokines, or proteases; and (iv) induce de-adhesion or support states of “intermediate adhesion” (i.e., a condition characterized by the reorganization of F-actin stress fibers to dismantle focal adhesions). Consequently, the group of matricellular proteins expanded and now includes additional TSPs (2–5), tenascins (R, W, X, Y), osteopontin, and members of the CCN family (discussed in more detail below; Murphy-Ullrich and Sage, 2014). The role of matricellular proteins has been mostly studied in wound healing, cancer, and the production of connective tissue. Some of these proteins, however, have also been studied in the nervous system. The classic matricellular proteins TSP-1 and SPARC were shown to accelerate synaptogenesis when released by astrocytes (Christopherson et al., 2005; Jones et al., 2011). Osteopontin expression increases after ischemic or mechanical brain injury and likely contributes to regenerative processes (Yan et al., 2009; van Velthoven et al., 2011; Plantman, 2012). The use of TN-C knockout mice revealed that TN-C is important for neuromuscular junction formation and plasticity (Cifuentes-Diaz et al., 1998), hippocampus and cortex structure and electrical properties (Irintchev et al., 2005; Gurevicius et al., 2009), hippocampal and cerebellar plasticity (Evers et al., 2002; Andjus et al., 2005), olfactory detection (de Chevigny et al., 2006) and learning and memory (Strekalova et al., 2002). TN-C also positively regulates neurite outgrowth (Rigato et al., 2002; Michele and Faissner, 2009). Similarly, TN-R was shown to regulate multiple processes in the nervous system, including neurogenesis (Xu et al., 2014), neuronal migration (Saghatelyan et al., 2004), axon navigation and neurite growth (Becker et al., 2003, 2004; Zacharias and Rauch, 2006), the formation of perineuronal nets (Brückner et al., 2000; Morawski et al., 2014), and neuron excitability and plasticity (Saghatelyan et al., 2001; Nikonenko et al., 2003; Gurevicius et al., 2004). In contrast, the CCN protein family received relatively little attention from neurobiologists. Nonetheless, CCN matricellular proteins are expressed in the nervous system and some examples exist to confirm their importance for the proper development and function of the nervous system.

## CCN Family

**Figure 1:**
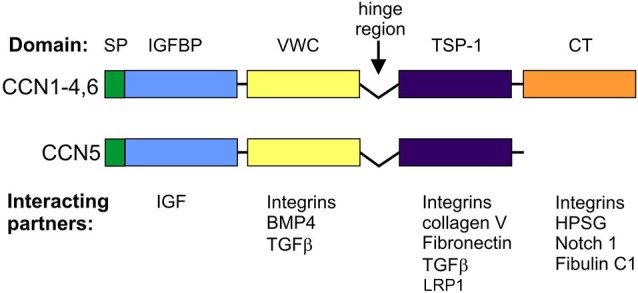
**Domain structure and canonical interactions of CCN family members**.

The CCN gene family (Cyr61/CTGF/NOV) consists of six members: *CCN-1* (*Cyr61*), *CCN-2* (*CTGF*), *CCN-3* (*NOV*), *CCN-4* (*WISP1*), *CCN-5* (*WISP2*), and *CCN-6* (*WISP3*). The family name was derived from early nomenclature for the first three identified proteins. These names often reflect the history of the discovery of particular CCN proteins or an initial view of their functions but may not necessarily reflect the current state of knowledge. For example, *CCN1* (*Cyr61)* was identified as 61st gene among genes, the expression of which was induced by trophic factors, e.g., epidermal growth factor (EGF) in fibroblasts (Lau and Nathans, 1987). Because of its mitogenic activity in connective tissue, *CCN2* was named connective tissue growth factor (*CTGF*; Bradham et al., 1991). *CCN3* was cloned as a gene that is overexpressed in nephroblastoma because of proviral insertion sites, and it was consequently named after this observation, i.e., Nephroblastoma overexpressed (*NOV*; Joliot et al., 1992). *CCN4* and *CCN5*, initially called expressed in low-metastatic type 1 cells (*ELM-1*; Hashimoto et al., 1998) and card-only protein 1 (*rCOP-1*; Zhang et al., 1998), respectively, were later identified as genes that were upregulated in response to Wnt-1 and consequently renamed WNT-inducible signaling pathway protein 1 (*WISP1*) and *WISP2* (Pennica et al., 1998). Finally, *CCN6* was identified as a *WISP1* homolog and named *WISP3*. This diversity of names within the CCN gene family (Table 1) became a major source of confusion and difficulty in attempts to follow research progress in the CCN field. Therefore, after the first international workshop on the CCN Family of genes, CCN researchers proposed a unification of the nomenclature for CCN family members (Brigstock et al., 2003); see also http://ccnsociety.com/ccn_nomenclature/index.html), which will be used hereinafter in the present review (Table 1). CCN family genes encode relatively small proteins (~40 kDa) that share ~40–60% similarity in their primary structure. Their most prominent common features are their functional domain composition and conservation of 38 cysteins through the peptide (Figure 1). Consequently, CCN proteins have several similarities in their molecular mechanisms of action and types of regulated cellular processes (e.g., cell adhesion, migration, proliferation, survival, and differentiation). However, the activity of particular CCN members may vary, depending on the cellular/tissue context (see below). Several extensive reviews have been published on the molecular biology of CCN proteins and their function in physiology and pathology (Leask and Abraham, 2006; Holbourn et al., 2008; Chen and Lau, 2009; Katsube et al., 2009; Jun and Lau, 2011; Perbal, 2013).

**Table 1:** **Alternative names and receptors of CCN proteins**.

**CCN1**
Alternative names: Cyr61^1^, CTGF-2, IGFB10, IGFB-rP4
Receptors: α_6_β1^2^, α_*V*_β_3_, α_*M*_β_2_, α_2_β_1_, α_*V*_β_5_, α_*D*_β_2_, α_*2b*_β_3_, HSPG
**CCN2**
Alternative names: CTFG, IGFBP8, IGFBP-rP2, HBGF0.8, ecogenin, FISP12
Receptors: α_*V*_β_3_, α_6_β_1_, α_5_β_1_, α_*M*_β_2_, TrkA, p75^NTR^, HPSG, LRP-1, LRP-6
**CCN3**
Alternative names: NOV, NOVH, IGFBP9, IGFBP-rP3
Receptors: α_*V*_β_3_, α_6_β_1_, α_5_β_1_, α_*v*_β_5_, Notch
**CCN4**
Alternative names: Wisp-1, Elm-1
Receptors: α_*v*_β_5_ (Hou et al., 2013; Stephens et al., 2015); α_*V*_β_3_ or β_1_ (Stephens et al., 2015), α_5_β_1_ (Liu et al., 2013)
**CCN5**
Alternative names: Wisp-2, CTGF-L, CTGF-3, HICP, Cop-1
Receptors: β_1_ integrin (Ohkawa et al., 2011), α_*v*_β_3_ (Myers et al., 2014), α_6_β_1_ (Haque et al., 2015)
**CCN6**
Alternative names: Wisp-3
Receptors: β1 integrin (Batmunkh et al., 2011), ανβ_5_ (Schütze et al., 2007)

*Abbreviations: ^1^Cyr61 cystein rich 61, CTGF—connective tissue growth factor, IGFB IGFB-rb—IGFB-related protein, HBGF—heparin-binding growth factor, FISP12 fibroblast-inducible secreted protein, NOV—nephroblastoma overexpressed, Wisp—Wnt-inducible secreted protein, Elm-1 expressed in low metastatic cells, HICP—heparin-induced CCN-like protein, Cop-1 card only protein 1. ^2^if not stated otherwise please refer to Jun and Lau (2011) for more detailed information*.

### Structure, Molecular Interactions, and Basic Modes of Action of CCN Proteins

CCN proteins are structurally very similar. With the exception of CCN5, CCNs contain four functional domains that are preceded by an export signaling peptide at their N-terminus, namely insulin-like growth factor binding protein (IGFBP), a von Willebrand factor type C repeat (VWC), thrombospondin type-1 repeat (TSP-1 or TSR), and a cystein knot-containing domain (CT; Figure 1). The last of these is missing in CCN5. IGFBP and VWC constitute the N-terminal half of CCN proteins, which is separated from the C-terminal half that contains TSP-1 and CT by a “hinge” region. Additional forms of CCNs, which have different activities from full-length proteins, can be produced by proteolysis (Brigstock et al., 1997; Steffen et al., 1998) or alternative splicing (Perbal, 2009). Each of the CCN protein domains is responsible for interactions with different sets of molecules (Figure 1). Consequently, CCN proteins bridge a variety of cell surface receptors, signaling molecules, and the ECM. Thus, modular composition, which is unique for the CCN family, explains the functional distinctiveness of CCNs from other proteins that contain one of the domains but raises the issue of what makes them functionally different from each other. Three-dimensional structure modeling revealed subtle changes in electrostatic surfaces that might be responsible for the differential interactions of particular CCN family members (Holbourn et al., 2008). However, most experimental evidence points to other sources of diversity, namely differential, often non-overlapping CCN expression patterns and different sets of receptors and/or proteins that interact with CCNs and are expressed by target cells.

CCN proteins have two major modes of action: (i) interactions with cell surface receptors; and (ii) interactions with receptor ligands. To date, the best studied are the cellular effects of CCN protein interactions with cell adhesion receptors [i.e., integrins and heparan sulfate proteoglycans (HSPGs); Table 1]. At the molecular level, such binding results in the cellular context-specific activation of signaling cascades, including extracellular signal-regulated kinases (ERKs), phosphoinositide 3-kinase (PI3K), and small GTPases of the Rho family (Leask and Abraham, 2006; Chen and Lau, 2009; Jun and Lau, 2011). This in turn leads to myriad cellular responses, of which changes in gene expression and cytoskeleton dynamics have been the most extensively studied. Some non-canonical outcomes (e.g., sustained reactive oxygen species production and cell senescence in response to CCN1-induced Rac1 activation) have also been described (Chen et al., 2007). One of the characteristic futures of CCN members is an ability to interact with various integrins (Table 1). Specific CCN proteins can bind different integrins, triggering dissimilar cellular responses. For example, in fibroblasts, CCN1 interactions with α_6_β_1_ stimulate cell adhesion, whereas its binding to α*_v_*β_3_ results in the induction of DNA synthesis. Moreover, the binding sites for different β-integrins do not overlap; therefore, simultaneous interactions with two integrins and a combined outcome are possible. However, cellular responses even to the same CCN protein-integrin couple may vary depending on the cell type (see below).

Integrins and HSPGs are currently the best described CCN receptors, but they are not the only ones. For example, the adhesion of rat hepatic stellate cells to a CCN2-coated surface requires binding to low-density lipoprotein receptor-related protein 1 (LRP-1) and HSPG (Gao and Brigstock, 2003). Binding to LRP-6 is likely responsible for the inhibitory effects of CCN2 on the canonical Wnt pathway in developing *Xenopus laevis* embryos. CCN2 binds tyrosine kinase receptor-A (TrkA) and p75 neurotrophin receptor (p75^NTR^; Wahab et al., 2005), whereas Notch is a receptor for CCN3 (Sakamoto et al., 2002; Minamizato et al., 2007). In addition to receptor binding, CCNs interact with receptor ligands and modulate their binding affinity or bioavailability. In the former case, the most thoroughly described interactions include those between CCNs and bone morphogenetic proteins (BMPs), which has an inhibitory effect, and between CCNs and transforming growth factor β (TGFβ), which has an activational effect (Abreu et al., 2002). CCN2 and CCN1 bind vascular endothelial growth factor (VEGF) and basic fibroblast growth factor (bFGF), respectively, and modify their bioavailability (Chen and Lau, 2009).

### General Functions of CCN Proteins

Detailed descriptions of the functions of CCN proteins have been provided in several reviews (Leask and Abraham, 2006; Holbourn et al., 2008; Chen and Lau, 2009; Katsube et al., 2009; Jun and Lau, 2011; Perbal, 2013). The diversity of CCNs’ modes of action results in a variety of cellular responses to CCN proteins. CCNs have been shown to regulate often converse cellular processes (e.g., cell proliferation/differentiation, cell death/survival, and cell adhesion/migration; for review see Jun and Lau, 2011; Table 2). Unsurprising is that different CCNs regulate opposite cellular processes. For example, CCN1, CCN2, and CCN3 are considered positive regulators of adhesion, whereas CCN5 and CCN6 are considered negative regulators (for review see Jun and Lau, 2011). However, opposing or very diverse cellular responses to the same CCN protein have also been reported. For example, CCN1 binds α_6_β_1_, αVβ_3_, α_2_Bβ_3_, and αMβ_3_ integrins and positively regulates the adhesion of fibroblasts and smooth muscle cells, activated endothelial cells, platelets, and monocytes and macrophages, respectively (for review see Jun and Lau, 2011). However, the binding of CCN1 to αVβ_5_ and α_6_β_1_ induces the migration of fibroblasts and smooth muscle cell chemotaxis, respectively (for review see Jun and Lau, 2011). As mentioned above, the binding of CCN1 to α*_v_*β_3_ integrin in fibroblasts does not affect adhesion/migration but rather regulates DNA synthesis. This example shows that CCN1 can have pleiotropic activities, depending on the cell type, functional/developmental cell status, and type of accessible receptors (e.g., integrins). The same is true for other CCN proteins (for review see Jun and Lau, 2011; Table 2).

**Table 2:** **Cellular functions of CCN proteins**.

**CCN1**
Cell adhesion^1^ (+)^2^, cell migration (+), DNA synthesis/proliferation (+), survival (+), apoptosis (+), differentiation (+), senescence (+)
**CCN2**
Cell adhesion (+), cell migration (+), survival (+), apoptosis (+)
**CCN3**
Cell adhesion (+), cell migration (+/−), DNA synthesis/proliferation (−/+), survival (+), apoptosis (+)
**CCN4**
Cell adhesion (+), (Liu et al., 2013; Stephens et al., 2015), migration (+) (Liu et al., 2013), proliferation (+) (Liu et al., 2013), survival (+) (Hou et al., 2013; Schlegelmilch et al., 2014)
**CCN5**
Cell migration (−), DNA synthesis/proliferation (−) (Haque et al., 2015), survival (+) (Ohkawa et al., 2011)
**CCN6**
Proliferation (+) (Batmunkh et al., 2011; Fang et al., 2014), migration (+) (Fong et al., 2012; Fang et al., 2014), survival (Huang et al., 2010)

*^1^if not stated otherwise please refer to Jun and Lau (2011) for more detailed information. ^2^+ positive effect, −negative effect, −/+ either positive or negative effect depending on cellular context*.

In addition to the intrinsic properties of a cell, the surrounding environment/tissue can also modulate responses to a particular CCN. Therefore, a deeper understanding of the physiological roles of CCNs and their links to human diseases became possible only with *in vivo* models. These *in vivo* studies point to a primary role for CCN family members in angiogenesis and cardiovascular and skeletal development. For example, CCN1, CCN2, and CCN3 stimulate *in vivo* blood vessel growth, whereas *CCN1* knockout (*CCN1*^−/−^) embryos die from placental vascular deficiency (Mo et al., 2002). The surviving embryos exhibit atrioventricular septal defects. *CCN1*^+/–^ mice are viable, but 20% of the adult mice have ostium primum atrial septal defects (Mo and Lau, 2006). Also *CCN2*-deficient mice have defects in vasculature remodeling (Hall-Glenn et al., 2012). CCN2, CCN3, CCN4, and CCN6 are mostly known for their importance in bone formation and/or remodeling. *CCN2* knockout mice exhibit skeletal deformations, reduced ossification, and the expansion of mineralizing cartilage zones. In *CCN3* knockout mice, Matsushita et al. (2013) observed accelerated bone regeneration. A study of transgenic mice that overexpress CCN4 in preosteoblasts reported that CCN4 stimulates osteogenesis (Ono et al., 2011). Finally, mutations in *CCN6* were repeatedly found in progressive pseudorheumatoid dysplasia, an autosomal recessive disorder (Hurvitz et al., 1999). Surprisingly, however, *CCN6* knockout mice have no obvious bone phenotype (Kutz et al., 2005; Hann et al., 2013), whereas some defects that may be relevant to human disease were observed in zebrafish morphants (Nakamura et al., 2007). In addition to the major research areas described above, CCNs have been extensively studied in the context of proper wound healing (Jun and Lau, 2011). Mounting evidence also indicates links between CCNs and tumorigenesis (Jun and Lau, 2011). In contrast, knowledge concerning the role of CCNs in the nervous system is relatively lagging. Part of the problem is that full, nonconditional knockout results in embryonic (*CCN1*, *CCN5*) or perinatal (*CCN2*) death. Often such early lethality precludes studies on several tissue/organ functions, including in the nervous system. In cases of CCN knockout animals that survive embryonic development, no apparent nervous system problems were reported, but usually deeper analyses in this regard have not been performed because different authors have different major interests (e.g., bone development). Another reason for the low recognition of CCNs’ roles in the nervous system stems from scattering of relevant information. To date, a comprehensive summary of the current state of knowledge concerning CCN expression patterns, the involvement of CCNs in neuronal development and physiology, and their links to nervous system pathology has been lacking.

## Expression and Functions of CCN Family Members in the Nervous System

Major research on CCN proteins has not focused on the nervous system, but for some CCNs, the central nervous system (CNS) is one of the major sites of expression. This observation supports the hypothesis of a possible role for the CNN protein family in different aspects of CNS physiology and pathology. In fact, the role of some CNN family members in the CNS has already been demonstrated, and the number of reports concerning this issue is constantly growing.

### CCN Expression in the Mammalian Central Nervous System

CCN proteins are expressed in non-neuronal tissues mainly during highly dynamic processes that involve tissue remodeling. CCN expression is thus observed during embryonic development and under pathological conditions (e.g., inflammation, injury, and cancer). Similarly, in the CNS, the expression of CCNs can be induced under such conditions. For example, both CCN1 and CCN2 were elevated in gliomas and glioblastomas (Pan et al., 2002; Xie et al., 2004a,b). However, CCN proteins are also expressed in the properly developing and adult CNS. Although knowledge of the pattern of CCN expression during embryogenesis and postnatal development and in the adult CNS is incomplete and restricted mainly to CCN1, CCN2, and CCN3, the number of studies that are investigating this issue is growing. Additional information concerning CCN expression patterns in the rodent and human brain can be extracted from large databases, including the collection of Allen Brain Atlases (ABA), e.g., Allen Mouse Brain Atlas (Lein et al., 2007)^1^ and Allen Developing Mouse Brain Atlas^2^ for *in situ* hybridization data, BrainStars* (BS*) for microarray data (Kasukawa et al., 2011)^3^, and the Human Protein Atlas (HPA) for immunohistochemistry data (Uhlén et al., 2015).^4^ The major advantages of these databases, which resulted from large-scale projects, are standardized procedures, reagents, and biological material. In this review, we use this knowledge to discuss previously published data and identify gaps in the literature.

#### CCN1 Expression in the Developing and Adult Central Nervous System

**Figure 2:**
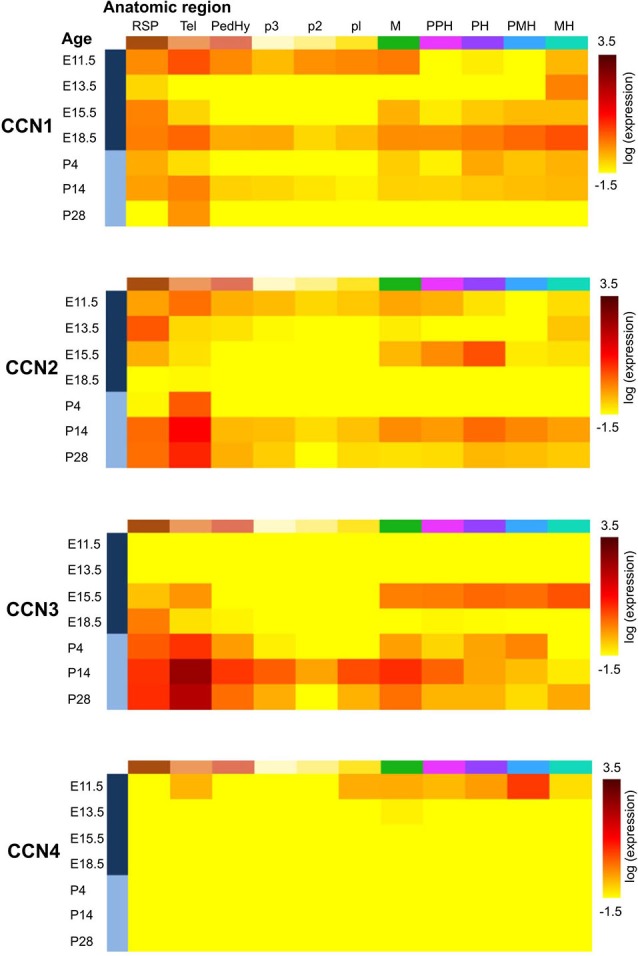
**Expression of CCN1–4 genes in the developing mouse brain according to the Allen Developing Mouse Brain Atlas [© 2013 Allen Institute for Brain Science**. **Allen Developing Mouse Brain Atlas (Internet)]**. Gene expression summary for selected brain structures were downloaded from http://developingmouse.brain-map.org/ and are expressed as a heatmap of expression energy (for more details please refer to http://help.brain-map.org/display/devmouse/In+Situ+Hybridization+(ISH)+Data#InSituHybridization(ISH)Data-CorrelationSearch). Heatmaps source: CCN1: http://developingmouse.brain-map.org/gene/show/15780; CCN2: http://developingmouse.brain-map.org/gene/show/13996; CCN3: http://developingmouse.brain-map.org/gene/show/17900; CCN4: http://developingmouse.brain-map.org/gene/show/22159. RSP—rostral secondary prosencephalon; Tel—telencephalic vesicle; PedHy—peduncular (caudal) hypothalamus; p3 prosomere 3; p2 prosomere 2; p1 prosomere 1; M—midbrain; PPH—prepontine hindbrain; PH—pontine hindbrain; PMH—pontomedullary hindbrain; MH—medullary hindbrain (medulla).

**Figure 3:**
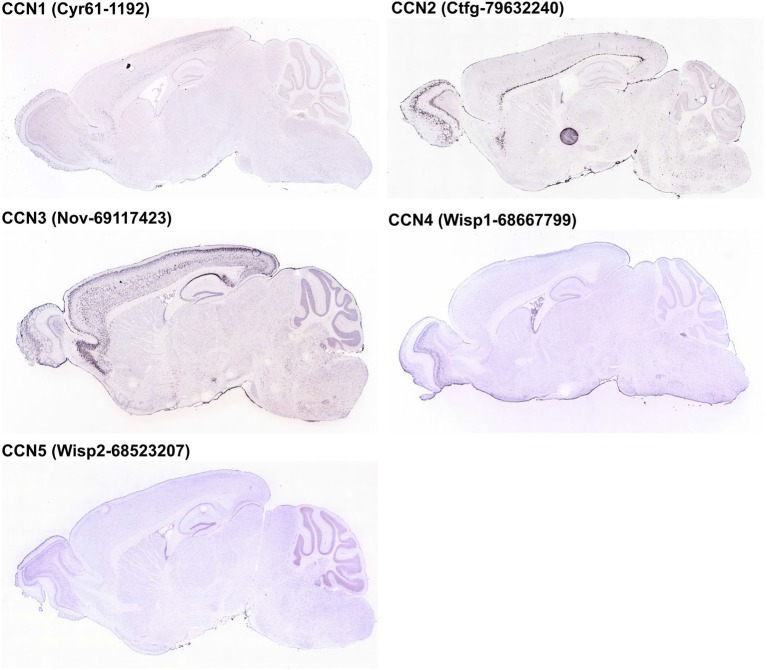
**Expression of CCN1–5 genes in the adult mouse brain according to the Allen Brain Atlas [© 2014 Allen Institute for Brain Science. Allen Mouse Brain Atlas (Internet)].** Images of brain sections were downloaded from http://mouse.brain-map.org/. Experiment name is given in parentheses.

**Figure 4:**
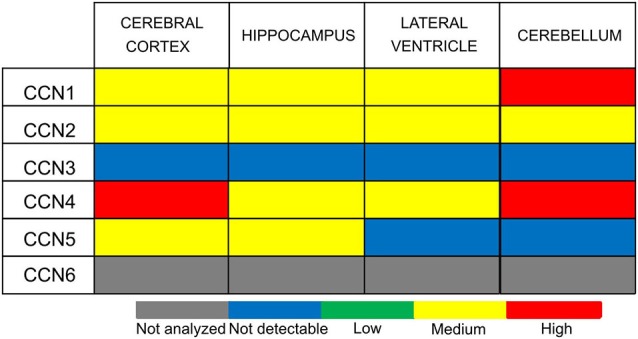
**CCN protein abundance in the selected human brain regions according to the Human Protein Atlas (HPA)**.

*CCN1* mRNA has been detected in different areas of the developing and adult human and rat brain and in rat hippocampal and cortical neurons cultured *in vitro* (Albrecht et al., 2000; Malik et al., 2013). Interestingly, *CCN1* mRNA levels in the rat hippocampus were higher during embryonic development and dropped postnatally, suggesting that this protein may play an important role during embryonic brain development (Malik et al., 2013). A similar developmental trend in *CCN1* expression was observed in cultured hippocampal neurons (Malik et al., 2013). An analysis of ABA data showed fluctuations of *CCN1* mRNA amount in embryonic brain but confirmed higher *CCN1* mRNA levels in the mouse embryonic day 18 (E18) brain compared with early postnatal weeks. The ABA suggests partial expression recovery in the cortex from postnatal day 28 (P28) onward (Figure 2). In the adult brain (P56) the ABA provides evidence for *CCN1* mRNA in deeper cortical layers (Figure 3). BrainStars* provides evidence of *CCN1* mRNA in the adult mouse CNS, with the highest levels in the retina. In the adult human brain, *CCN1* mRNA was detected throughout the CNS, with the strongest expression in the spinal cord, frontal, temporal, and occipital cortices, hippocampus, and caudate nucleus (Albrecht et al., 2000). This observation appears to be confirmed by the HPA, in which mild CCN1 protein levels were present in the cortex and hippocampus, but the highest levels were detected in the cerebellum (Figure 4). An analysis of the cell specificity of immunohistochemical signal showed that CCN1 protein can be detected mostly in neurons, whereas its expression in glia is lower or undetectable (Table 3).

**Table 3:** **Cellular specificity of CCN protein expression in selected regions of the CNS according to the Human Protein Atlas**.

	Cerebral cortex	Hippocampus	Lateral ventricle	Cerebellum
**CCN1**	endothelial cells—medium glial cells—low neuronal cells—medium neuropil—medium	glial cells—not detected neuronal cells—medium	glial cells—low neuronal cells—medium	cells in granular layer—medium cells in molecular layer—low Purkinje cells—high
**CCN2**	endothelial cells—medium glial cells—medium neuronal cells—medium neuropil—medium	glial cells—low neuronal cells—medium	glial cells—low neuronal cells—medium	cells in granular layer—medium cells in molecular layer—medium Purkinje cells—medium
**CCN3**	endothelial cells—not detected glial cells—not detected neuronal cells—not detected neuropil—not detected	glial cells—not detected neuronal cells—not detected	glial cells—not detected neuronal cells—not detected	cells in granular layer—not detected cells in molecular layer—not detected Purkinje cells—not detected
**CCN4**	endothelial cells—high glial cells—medium neuronal cells—medium neuropil—medium	glial cells—medium neuronal cells—medium	glial cells—medium neuronal cells—medium	cells in granular layer—medium cells in molecular layer—medium Purkinje cells—high
**CCN5**	endothelial cells—not detected glial cells—not detected neuronal cells—medium neuropil—not detected	glial cells—not detected neuronal cells—medium	glial cells—not detected neuronal cells—not detected	cells in granular layer—not detected cells in molecular layer—not detected Purkinje cells—not detected
**CCN6**	not analyzed	not analyzed	not analyzed	not analyzed

Our data from *in vitro* hippocampal neuron cultures showed that CCN1 expression can be modulated by various stimuli [e.g., brain-derived neurotrophic factor (BDNF), insulin, and bicuculline], both in developing and “mature” neurons. The effects of bicuculline suggest a role for enhanced excitatory transmission in the regulation of *CCN1* mRNA levels. Similar results were obtained in organotypic hippocampal slices (Iacono et al., 2013). *In vivo*, *CCN1* mRNA level increases with decreases in excitatory transmission that are caused by *N*-methyl-D-aspartate receptor inhibition (Ito et al., 2007). Nevertheless, the mechanism behind this phenomenon might be indirect and involve increases in the activation of other neurotransmitter systems. Elevations of *CCN1* mRNA were also observed upon methamphetamine administration (Ito et al., 2007), administration of the dopamine receptor agonist clozapine (Sakuma et al., 2015), and the activation of muscarinic acetylcholine receptors (Chung and Ahn, 1998; Albrecht et al., 2000).

#### CCN2 Expression in the Developing and Adult Central Nervous System

Similar to *CCN1*, *CCN2* is also expressed in different parts of the CNS and rat primary neuron cultures *in vitro* (Albrecht et al., 2000), and its levels are regulated developmentally. *CCN2* mRNA is present in neural tissue in developing mouse embryos already at embryonic day 9.5 (Ivkovic et al., 2003). Williams et al. (2007) reported presence of *CCN2* mRNA in olfactory bulb at embryonic days 14, 17.5 and at postnatal day 3. In the adult brain presence of both *CCN2* mRNA and CCN2 protein was reported in the hippocampus (Hertel et al., 2000) and olfactory bulb (Williams et al., 2007). The ABA shows moderate levels of *CCN2* mRNA between E11.5-E15.5 and a complete lack of *CCN2* expression just before birth. However, *CCN2* mRNA levels gradually increase from P4 until adulthood (Figure 2). Such dynamic regulation of *CCN2* expression in early postnatal development was studied in detail in the rodent olfactory bulb. *CCN2* mRNA in the glomerular and mitral cell layers could be detected around P5. In the glomerular layer, *CCN2* mRNA level remained stable throughout postnatal development and in the adult brain, whereas it gradually decreased in the mitral cell layer and was barely detectable already on P12 (Khodosevich et al., 2013). In the adult rat brain, *CCN2* mRNA showed a highly selective distribution pattern in the forebrain, with strong labeling that was restricted to specific regions of the olfactory bulb, endopiriform nucleus, and supracallosal layer in the cerebral cortex. Particularly strong staining was observed in large neurons of layer VI in the cerebral cortex (Heuer et al., 2003). In the adult mouse brain, *CCN2* mRNA could be detected in layer VI of the cortex and mitral cell and glomerular layers of the olfactory bulb (Khodosevich et al., 2013). These observations are consistent with the ABA (Figure 3) and BS* data. Presence of *CCN2* mRNA was also shown throughout the adult human CNS, including different brain regions and the spinal cord. In fact, the *CCN2* expression pattern is similar to *CCN1*, with the exception that tissue levels of *CCN2* transcripts were lower in the hippocampus, caudate nucleus, and corpus callosum (Albrecht et al., 2000). In the healthy human brain, CCN2 protein was predominantly detected in neurons and partially in subtypes of glial cells (Schwab et al., 2000, 2001; Table 3). The HPA also suggests the presence of CCN2 protein in glial cells, especially in cortical areas (Table 3). Supporting further glial expression of *CCN2*, its expression has been detected in rat CNS astrocytes and tanycytes (Kondo et al., 1999). In the normal human spinal cord, the low expression of *CCN2* was observed in cells with astroglial morphology, particularly in white matter (Spliet et al., 2003). In the senile human brain, exclusively *CCN2* neuronal expression was found throughout different brain areas, particularly in entorhinal layer pre-alpha neurons and cortical pyramidal cells, both in somatodendritic and axonal compartments (Ueberham et al., 2003). Expression of *CCN2* also appears to be regulated by neuronal activity. Khodosevich et al. (2013) demonstrated that CCN2 protein level is dynamically regulated in response to changes in olfactory input.

#### CCN3 Expression in the Developing and Adult Central Nervous System

*CCN3* is also expressed in the developing and adult CNS. In fact, the CNS appears to be the major site of *CCN3* expression during development compared with other tissues. This was demonstrated in chick embryos (Joliot et al., 1992) and human embryos in the first trimester of embryogenesis, in which *CCN3* mRNA and CCN3 were most abundantly present in motor neurons and the floor plate of the spinal cord (Kocialkowski et al., 2001). *CCN3* expression undergoes dynamic changes during development, suggesting that CCN3 protein might play a role in maintaining or establishing specific brain functions. The abundance of *CCN3* mRNA increases throughout the postnatal period during rat brain development. Increases in amount of *CCN3* mRNA and CCN3 protein were detected in the developing rat brain after birth, with a pronounced peak between P15 and P150 (Su et al., 2001). A similar increase seems to occur in the developing murine brain according to the ABA (Figure 2) through a majority of brain areas and the highest levels of *CCN3* mRNA are present in olfactory bulb, cortex and CA1 field of hippocampus (Figure 3). Intriguingly, the ABA also reports relatively low levels of *CCN3* mRNA in the cerebellum. But *CCN3* mRNA and CCN3 protein were detected in the rat cerebellum during the postnatal period, with the most prominent increase from P7 to P14 (Le Dréau et al., 2010b). In the human brain during early developmental stages, *CCN3* mRNA was mainly observed in somatomotor neurons in the lower CNS. At later stages, however, *CCN3* was expressed in the higher CNS (Su et al., 1998). High levels of *CCN3* mRNA were reported in the cortex, hippocampus, amygdala, and spinal cord (Albrecht et al., 2000). Surprisingly, the HPA reveals no CCN3 protein expression in the analyzed areas (Figure 4).

*CCN3* expression is particularly observed in neurons. For example, presence of *CCN3* mRNA was shown in rat neuronal cultures *in vitro* (Albrecht et al., 2000) while CCN3 protein was detected in rat cerebellar Purkinje cells (Su et al., 2001; Le Dréau et al., 2010b), and neurons of dorsal root ganglia and the dorsal horn of the rat spinal cord (Kular et al., 2012). However, CCN3 immunoreactivity was also observed in astrocytes in the rat cerebral cortex, corpus callosum, and hippocampus (Le Dréau et al., 2010a). *CCN3* mRNA and CCN3 protein are also expressed in specific structures in the developing chicken eye, including the lens, ciliary body, optic nerve, pecten, and retina (Laurent et al., 2012).

#### CCN4, CCN5, and CCN6 Expression in the Central Nervous System

In contrast to CCN1–3, very little is known about CCN4, CCN5, and CCN6 expression patterns. The data that are available are also somewhat contradictory. A study of *CCN4*, *CCN5*, and *CCN6* mRNA levels in human embryonic and adult tissues showed no expression in the brain (Pennica et al., 1998), but other studies do not fully support this finding. Indeed, during rat embryonic development, *CCN4* mRNA presence was reported exclusively in osteoblasts and osteoblastic progenitor cells of the perichondral mesenchyme (French et al., 2004). The ABA also does not reveal the presence of *CCN4* mRNA during development after E11.5 (Figure 2). But, both the ABA and BS* suggest that *CCN4* mRNA is present in the adult murine brain, although at relatively low levels, with the highest expression in the olfactory bulb (ABA, BS*) and retina (BS*; Figure 3). Also, the HPA shows presence of CCN4 protein in the adult human cerebral cortex and cerebellum (Figure 4), with the highest expression levels in cortical endothelial cells and Purkinje neurons (Table 3). Additionally, CCN4 protein was shown to be expressed in rat primary neurons during oxygen-glucose deprivation (Wang et al., 2012). *CCN5* mRNA, according to the ABA, is not present in P56 murine brain (Figure 3). But the BS* reports low to moderate *CCN5* mRNA levels throughout the adult mouse CNS, with the highest expression in the retina. Moreover, Ohkawa et al. (2011) detected some *CCN5* mRNA in the cerebellum and spinal cord of 15 week old mice. In contrast, CCN5 immunostaining was reported in fetal mouse brain (E12–16) but not human brain (4 months; Jones et al., 2007). Cytoplasmic and nuclear CCN5 immunostaining was also reported in cortical neurons in the adult rat (Gray et al., 2007). The presence of CCN5 protein in the adult cortex is supported by the HPA (Figure 4), which additionally provides evidence of its hippocampal expression. Virtually nothing is known for CCN6. No data are reported concerning its expression in either the ABA or HPA. Only a BS* microarray analysis detected almost equal amounts of CCN6 transcript in all 51 analyzed brain regions.

### Role of CCN Proteins in the Central Nervous System

CCNs can be detected in the CNS. Importantly, CCN levels dynamically change during development. This observation supports their possible role in CNS development and physiological functions. Here, we will discuss the available data that indicate the involvement of CCN proteins, mainly CCN1, CCN2, and CCN3, in CNS development and physiology.

#### CCN1

In 1998, CCN1 was suggested to play an important role in neuronal differentiation, in which it was induced by bFGF during the differentiation of immortalized hippocampal progenitor (H19–7) cells (Chung and Ahn, 1998). Indeed, we recently confirmed that CCN1 is required for proper development of the dendritic tree in rat hippocampal neurons *in vitro* and acts downstream of Ras, ERK, and PI3K. Moreover, CCN1 overexpression promoted dendritic branching, and this effect depended on β1-integrin (Malik et al., 2013).

CCN1 is secreted by retinal Muller glial (RMG) cells that are cultured *in vitro* in response to glial cell line-derived neurotrophic factor (GDNF) treatment, which is known to have pro-survival effects in the retina. Thus, the neuroprotective and pro-survival activity of CCN1 was studied in a mouse model of retinitis pigmentosa (Kucharska et al., 2014). In organotypic retinal cultures that were derived from these mice, CCN1 treatment increased the survival rates of photoreceptors. The authors showed that CCN1 activates the mitogen-activated protein kinase (MAPK)/Erk and Janus-associated kinase (JAK)/Stat pathways but not PI3K/Akt pathway in retinal explants from retinitis pigmentosa mice. Interestingly, in primary porcine cultures, CCN1 did not stimulate photoreceptors themselves but rather only RMG cells and retinal pigment epithelium (RPE) cells, suggesting that the protective effect on photoreceptors occurs indirectly. In disagreement with the results that were obtained in the retinitis pigmentosa model, CCN1 treatment in pure porcine RMG cultures stimulated not only the MAPK/Erk and JAK/Stat pathways but also the PI3K/Akt pathway, whereas it led to activation of the PI3K/Akt and MAPK/Erk pathways in RPE cultures.

The stimulation of muscarinic acetylcholine receptors (mAChRs) induced *CCN1* mRNA expression in primary neurons and the rat brain, in which *CCN1* mRNA was detected in cortical layers V and VI and thalamic nuclei. mAChRs modulate neuronal functions, including long-term potentiation and synaptic plasticity in neuronal circuits that are involved in learning and memory formation. The authors suggested a role for CCN1 in the cholinergic regulation of synaptic plasticity (Albrecht et al., 2000).

#### CCN2

Consistent with its expression pattern, CCN2 was shown to play an important role in the rodent olfactory bulb, where it acts as a proapoptotic factor that eliminates newborn neurons in an activity-dependent and locally restricted manner. CCN2 acts via glial-derived transforming growth factor β2 (TGF-β2) to promote the apoptosis of newly generated periglomerular interneurons in the glomerular layer (Khodosevich et al., 2013). CCN2 levels, which are dynamically regulated in response to changes in olfactory input, adjust the survival of postnatally born neurons in an odorant-specific fashion. Importantly, CCN2 levels decrease in the mouse olfactory bulb if the sensory input is suppressed (Khodosevich et al., 2013). In contrast, olfactory activity enhances *CCN2* expression specifically in odor-activated glomeruli in the olfactory bulb. This phenomenon has important behavioral consequences, and *CCN2* knockdown mice perform better in odorant detection and olfactory discrimination than controls.

As mentioned above, CCN2 binds TrkA and p75NTR, receptors that transduce neurotrophin signals (Wahab et al., 2005). The authors showed that CCN2 stimulates TrkA and induces its autophosphorylation. The nerve growth factor (NGF)-induced activation of TrkA is widely known to control neuronal cell survival and axonal growth (Kuruvilla et al., 2000; Atwal et al., 2003). Nonetheless, the physiological importance of CCN2’s influence on neurotrophin signaling in brain development, physiology, and disease remains to be evaluated.

#### CCN3

A role for CCN3 in brain development has also been reported. Specific patterns and dynamic changes in *CCN3* expression in the rat cerebellum (Le Dréau et al., 2010b) likely reflect its function in postnatal cerebellum development. Supporting this possibility, an *in vitro* study showed that CCN3 reduces cerebellar granule neuron precursor (GNP) proliferation that is induced by Sonic Hedgehog (SHH). SHH maintains GNPs in a proliferative state and delays their differentiation, resulting in a decrease in the proportion of postmitotic neurons. By counteracting SHH-driven effects, CCN3 reduces the proliferation of GNPs and consequently promotes their differentiation. This anti-proliferative action of CCN3 requires glycogen synthase kinase 3β (GSK3-β) activity. Moreover, CCN3 stimulates the migration and chemotaxis of cerebellar GNPs *in vitro* (Le Dréau et al., 2010b). The role of CCN3 in the retina has also been studied. *CCN3* is expressed in the chick retina during development in distinct cell types and is regulated by the Notch and BMP signaling pathways (Laurent et al., 2012). However, the ectopic expression of CCN3 had no effect on retinal development. These authors did not describe the effects of CCN3 depletion, and the possible role of CCN3 in the developing retina remains unclear (Laurent et al., 2012).

#### CCN5

A role for CCN5 in neuronal development has also been suggested. CCN5 was shown to enhance neurite outgrowth in Neuro2a cells. The underlying mechanism remains elusive, although the authors suggested the involvement of the CCN5-triggered, β1-integrin-dependent activation of Akt (Ohkawa et al., 2011). Thus, far no data are available concerning neuronal functions of CCN4 and CCN6. Most likely almost complete lack of data concerning role of CCN4, 5 and 6 in the nervous system reflects their low abundance in the nervous system and relatively poor repertoire of tools to reliably study their expression. Alternatively, those proteins may attract less attention when identified in large “omics” screens because of limited knowledge concerning their cellular functions.

## CNN Proteins and Nervous System Dysfunction

As in non-neuronal tissues, CCN proteins play a role in responses to injury in the CNS. This role has been particularly attributed to CCN2. CCN2 appears to be involved in gliosis and glia scar formation in response to different types of brain injury, such as trauma, cerebral infarction, and excitotoxic brain damage. After kainic acid-induced lesions of the CA3 area of the hippocampus (i.e., a model of excitotoxic brain damage), CCN2 protein was detected in the CA1, CA3, and dentate gyrus, mainly in neurons that mostly died on subsequent days. At later stages when repair processes are active, CCN2 was found extracellularly and in GFAP-positive astrocytes (Hertel et al., 2000).

CCN2 upregulation has also been observed in reactive gliosis adjacent to the site of mechanical injury and in brain tissue in stroke patients and following traumatic brain injury (Schwab et al., 2000, 2001). A role in neuroinflammation has also been suggested for CCN3. CCN3 protein was shown to be expressed in astrocytes and regulate astrocyte chemokine synthesis *in vitro* and *in vivo* (Le Dréau et al., 2010a). CCN3 was suggested to attenuate inflammatory pain (Kular et al., 2012). CCN3 protein expression decreased in dorsal root ganglia and the dorsal horn of the spinal cord in a rat model of inflammatory pain. Interestingly, intrathecal administration of CCN3 siRNA during early stages of an inflammatory pain model resulted in a significant increase in mechanical allodynia. In contrast, CCN3 treatment significantly attenuated mechanical allodynia in rats but had no effect on basal pain perception in control animals. As demonstrated both *in vitro* and *in vivo*, CCN3 influences matrix metalloproteinase (MMP) expression, which might contribute to the possible mechanism of CCN3’s involvement in inflammatory pain (Kular et al., 2012).

The role of several CCN proteins has been suggested in neurodegenerative disease, based largely on the observation that they are expressed in patients’ brains in both neurons and astrocytes. Notably, however, a causal role for CCN proteins in neurodegeneration has not yet been proven, and increased levels of CCNs might result from inflammation that accompanies neurodegeneration. CCN2 appears to be involved in the progression and persistence of astrogliosis in neurodegenerative diseases, including amyotrophic lateral sclerosis (ALS) and Alzheimer’s disease (AD). In AD patients’ brains, CCN2 protein was detected in the entorhinal cortex, hippocampus, and temporal cortex, and the signal was associated with neurofibrillary tangles and neurons that are associated with amyloid plaques (Ueberham et al., 2003). Moreover, CCN2 was detected in perivascular astrocytes and astrocytes that were associated with plaques (Ueberham et al., 2003). Another study showed that *CCN2* expression in the AD brain is correlated with the progression of clinical dementia in AD and amyloid plaques but not neurofibrillary tangle pathology. The authors suggested that CCN2 may play a role in the pathogenesis of AD by promoting amyloid β peptide levels. This hypothesis was supported by findings from a Tg2576 AD model in mice that were exposed to a diabetogenic diet. These mice developed insulin resistance, accompanied by elevations in CCN2 levels in the brain and the promotion of AD-type amyloid plaque burden (Zhao et al., 2005). The authors also found that CCN2 activated the MAPK and PI3K/Akt pathways in human H4-APP_751_ neuronal cells and suggested that this may lead to an increase in amyloid β peptide levels and contribute to AD pathology (Zhao et al., 2005).

*CCN2* expression was also linked to ALS. In normal human spinal cord, the low expression of CCN2 protein is observed in cells with astroglial morphology, particularly in white matter. CCN2 protein levels are increased in ALS cases. It can be detected in vimentin-positive reactive astrocytes and remaining motor neurons in the ventral horn in long-term-surviving patients (Spliet et al., 2003).

CCN4 has been linked to neurodegeneration protection. Treatment with CCN4 blocked primary neuronal injury and apoptosis during oxygen-glucose deprivation, and this effect depended on PI3K-Akt signaling (Wang et al., 2012).

## Conclusions and Perspectives

CCN proteins have not been studied vigorously or systematically in the nervous system, but their known functions in other tissues and organs support their potential involvement in the proper development and physiology of the nervous system. Such a role is also supported by a few discoveries of CCNs’ involvement in neuroprecursors proliferation, neuronal survival, and differentiation. Examples of nervous system pathologies that are linked to changes in CCN gene expression support the importance of CCNs in the nervous system. Yet, based on these few examples it would be premature to build a comprehensive model of CCN protein-dependent regulation of the nervous system development and physiology. As in case of other systems, it is very likely that the final outcome of CCN activities may depend on cell type and repertoire of receptors expressed by a given cell at a particular developmental/differentiation stage. What is more, similar to studies on skeletal development, studies in the context of the whole organism are key to understanding CCNs’ modulatory functions. Therefore, further progress in our understanding of the functions of CCNs in the nervous system will require the development of animal models using spatially and developmentally regulated knockouts of CCN family genes. Studies on the role of CCNs in the nervous system can accelerate in the future only with such models.

## Conflict of Interest Statement

The authors declare that the research was conducted in the absence of any commercial or financial relationships that could be construed as a potential conflict of interest.

## Footnotes

^1^http://www.brain-map.org

^2^http://developingmouse.brain-map.org

^3^http://www.brainstars.org

^4^http://www.proteinatlas.org
